# Correlation Between Gastric and Gallbladder Helicobacter pylori Infection in South Indian Patients Undergoing Cholecystectomy for Gallbladder Disease

**DOI:** 10.7759/cureus.92726

**Published:** 2025-09-19

**Authors:** Abheesh Hegde, Sajal Gupta, Syed F Ahamed, Renuka Mallipatel, Mary Dias, Vivek Rosario, Mallikarjun Patel, Anthony Rozario

**Affiliations:** 1 Urology, Father Muller Medical College, Mangalore, IND; 2 General Surgery, Vardhman Mahavir Medical College and Safdarjung Hospital, New Delhi, IND; 3 Infectious Diseases, Independent Consultant, Bangalore, IND; 4 Pathology, St. John's National Academy of Health Sciences, Bangalore, IND; 5 Microbiology, St. John's Medical College, Bangalore, IND; 6 Gastroenterology, St. John's Medical College, Bangalore, IND; 7 Surgery, St. John's National Academy of Health Sciences, Bangalore, IND

**Keywords:** biliary, cholecystectomy, cholelithiasis, gastroscopy, helicobacter pylori (h. pylori), inflammation

## Abstract

Background: *Helicobacter pylori* has been established as an etiological agent in gastric adenocarcinoma and mucosa-associated lymphoid tissue (MALT) lymphoma in the stomach. Recent reports have implicated this bacterium in the causation of benign and malignant gallbladder disease. We investigated the correlation between gallbladder and gastric mucosal *H. pylori* in patients with symptomatic cholelithiasis.

Aims: To estimate the prevalence of *H. pylori* colonization in the gallbladder and assess its correlation with gastric *H. pylori* in South Indian patients undergoing cholecystectomy.

Methods: A cross-sectional prevalence study was conducted at a tertiary care center in South India. A total of 49 consecutive patients undergoing cholecystectomy with clinical indications for preoperative gastroscopy were enrolled. Gastric mucosal biopsies were assessed using culture, rapid urease test, and DNA polymerase chain reaction (PCR). Gallbladder tissue was evaluated via culture, PCR, histology (modified Giemsa), and rapid urease test. Concordance between gastric and gallbladder positivity was analyzed.

Results: Out of 49 patients undergoing cholecystectomy, 12 (24.5%) were in the age group of 51-60 years. Females comprised 64.89% of the study population. Laparoscopic cholecystectomy was performed in 40 (81.91%) of the cases, and chronic cholecystitis was diagnosed in 43 (88%) patients. Among the 49 gastric specimens analyzed for *H. pylori*, all tested negative by rapid urease test and culture; however, 10 samples were positive for *H. pylori* by DNA PCR (10/49 gastric PCR positive; 20.4%, 95% CI: 10.2-33.7%). In contrast, all 49 gallbladder samples were negative for *H. pylori* by DNA PCR, culture, histopathology, and rapid urease test.

Conclusion: *H. pylori* was not detected in gallbladder tissue among South Indian patients despite a 20.4% prevalence in the gastric mucosa. These findings suggest no correlation between gastric and gallbladder *H. pylori* colonization in this cohort. Further multicentric studies are needed to evaluate regional microbiological and pathological variations.

## Introduction

Lord Moynihan described a gallstone as "a tombstone erected to the memory of organisms which lie dead within them." Marshall and Warren's work definitively recognized *Helicobacter pylori* as the causative agent of gastritis and peptic ulcer disease [1]. Since then, *H. pylori* has been linked to gastric adenocarcinoma and mucosa-associated lymphoid tissue (MALT) lymphoma. The prevalence of *Helicobacter* infection varies significantly across populations and countries [2]. Several species, including *H. cinaedi*, *H. fennelliae*, *H. canis*, *H. rappini*, *H. pullorum*, and *H. canadensis*, have been isolated from human intestinal tracts. According to a hypothesis that suggests *H. pylori* may relate to gallbladder cancer, similar to its role in gastric malignancies, it potentially contributes through chronic cholecystitis secondary to gallstones, leading to metaplasia, dysplasia, and carcinoma [3]. This suggests disease prevention through *H. pylori* eradication, and clinicians treating gastric *H. pylori* should consider evaluating gallbladder conditions. Gallbladder cancer incidence is highest among women in India, Pakistan, and Ecuador, driving interest in the bacterium's link to gallstone-related pathology [4].

Studies have shown inconsistent findings on the coexistence of *H. pylori *in the stomach and its link to other gastrointestinal diseases, including gallbladder infections, gastric cancer, and liver disorders. Studies indicate that the presence of *H. pylori* in both the stomach and gallbladder could explain variations in clinical presentations and outcomes [5]. Geographic and ethnic factors influence the role of *H. pylori *in gallbladder diseases. In India, gallbladder pathology is a major health burden, with the highest cancer rates in northern regions, while South India shows lower gallbladder cancer incidence and *H. pylori* prevalence [6]. This is the first study from South India using four detection methods to assess *H. pylori*. This study examined the relationship between gastric and gallbladder *H. pylori* colonization in South Indian patients undergoing cholecystectomy.

Aims and objectives

Primary Aim

To estimate the prevalence of *H. pylori* colonization in gallbladder tissue using multiple detection methods (polymerase chain reaction (PCR), culture, histology, and urease) in South Indian patients undergoing cholecystectomy.

Secondary Aims

To determine the prevalence of gastric *H. pylori* using PCR in the same cohort, to assess concordance between gastric and gallbladder colonization, and to describe the histopathological spectrum of gallbladder disease and associated patient demographics.

## Materials and methods

Sample size

**Figure 1:**
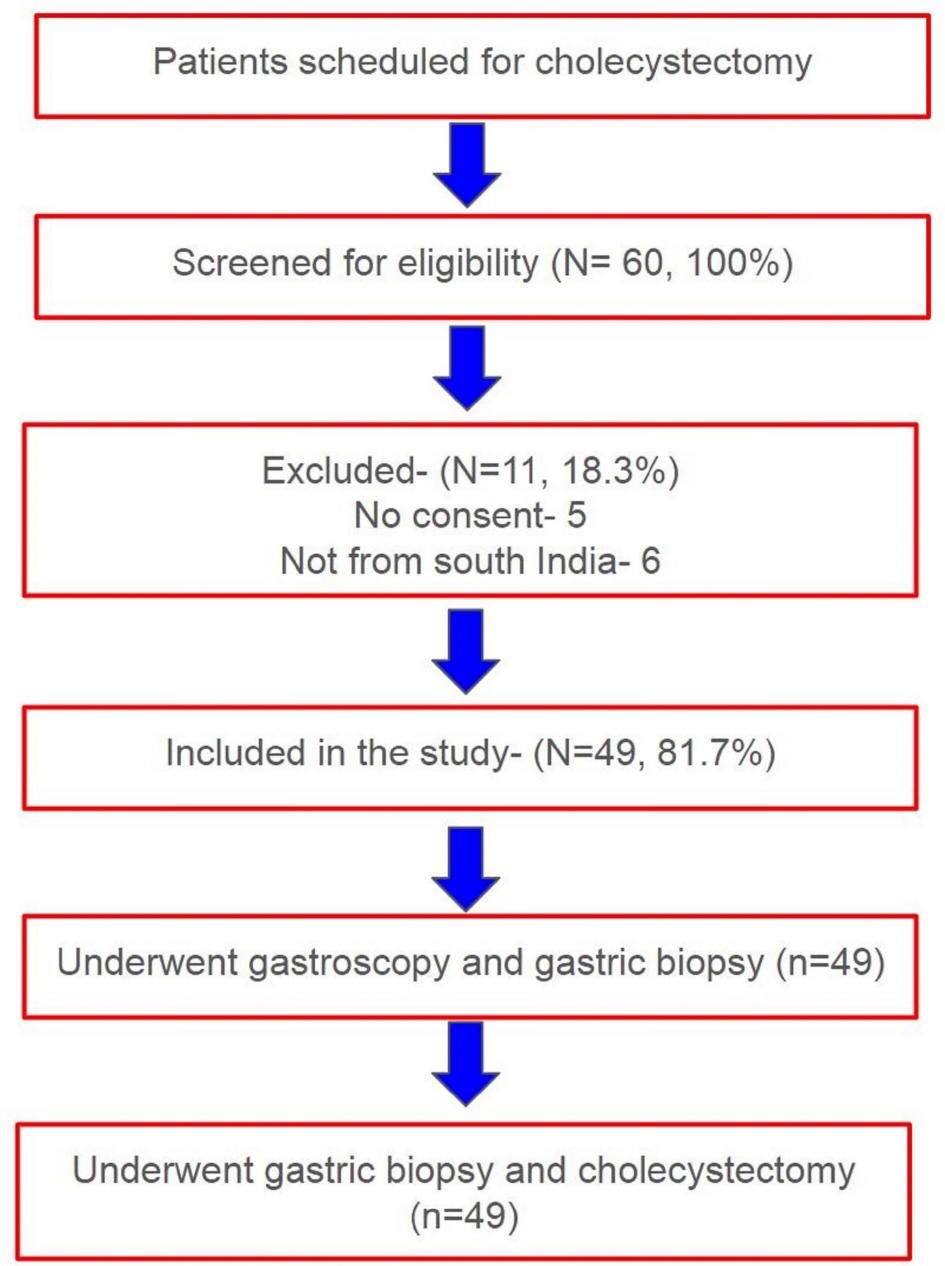
Patient flow diagram.

The study enrolled 49 patients who underwent cholecystectomy for symptomatic gallbladder disease over the course of one year at a tertiary care hospital. Institutional Ethics Committee approval was obtained prior to the commencement of the study (approval number: 116/2014). This was a cross-sectional prevalence study within a cholecystectomy cohort. The required sample size was determined using nMaster software (Department of Biostatistics at Christian Medical College (CMC), Vellore, India), based on findings from a study by Hegde et al. [5], aiming for 10% precision (Figure 1).

Inclusion criteria

The inclusion criteria included the following: adult patients undergoing elective or emergency cholecystectomy for gallbladder disease; originating from the South Indian states of Karnataka, Kerala, Tamil Nadu, and Andhra Pradesh; patients who underwent preoperative upper gastrointestinal endoscopy due to clinical indications such as suspected peptic ulcer, common bile duct (CBD) stones, or obstructive jaundice; provided written informed consent.

Exclusion criteria

The exclusion criteria included the following: patients from North India (to maintain regional homogeneity); patients unwilling to undergo preoperative gastroscopy; patients with a recent history (within one month) of *H. pylori* eradication therapy or recent antibiotic/proton pump inhibitor use (excluded by history).

Study protocol

Patient Enrollment and Sample Collection 

Patients undergoing cholecystectomy for symptomatic gallstone disease at St. John's Medical College Hospital, Bangalore, between March 2014 and March 2016, were prospectively enrolled. Inclusion criteria were age ≥18 years, clinical and radiological confirmation of gallstone disease, and willingness to participate with informed consent. Patients with prior *H. pylori* eradication therapy, antibiotic use within four weeks, or known malignancy were excluded [7].

Paired gastric biopsies (antrum and body) and gallbladder mucosal samples were collected at the time of surgery in sterile conditions. To reduce contamination, intra-gastroduodenal and intra-biliary samples were collected with changed instruments. Specimens were separated for culture, rapid urease test (RUT), histology, and PCR, and immediately taken in sterile containers packed on ice and sent to the microbiology laboratory.

Culture

Subsequently, the samples were homogenized and seeded onto plates of Columbia agar with 7% defibrinated sheep white blood cells (Difco) plus selective antibiotics (vancomycin, trimethoprim, polymyxin B, amphotericin B). Plates were incubated at 37°C for a maximum of seven days in a microaerophilic environment (5% O₂, 10% CO₂, and 85% N₂) produced using CampyGen gas pack kits (Oxoid, Thermo Fisher Scientific, Waltham, MA). *H. pylori* was characterized by classic colony morphology, Gram's staining, and positive oxidase, catalase, and urease tests.

Rapid Urease Test

RUT was conducted by using the commercially available kit for gastric biopsies and gallbladder mucosa. The tests were analyzed at one, six, and 24 hours. A positive result was defined as a color change from yellow to red/pink.

Histopathology

Biopsy and gallbladder tissues were fixed in 10% buffered formalin, processed through paraffin embedding, and hematoxylin & eosin, along with modified Giemsa stains, were applied. The slides were assessed by pathologists who were blinded to microbiological findings. A specimen was considered *H. pylori*-positive if spiral organisms were observed along the surface epithelium of the gastric antrum and body, or within the mucus layer. Negative classification was dependent on the absence of organisms in an appropriate tissue representation.

Polymerase Chain Reaction

DNA was obtained with the DNA Mini package. The ureA gene was targeted for PCR amplification by published primers: forward: 5’-AAGCTTTTAGGGGTGTTAGGGGTTT-3’; reverse: 5’-AAGCTTACTTTCTAACACTAACGC-3’.

PCR was carried out in a 25 µL reaction volume consisting of 2.5 µL of 10× buffer, 1.5 mM MgCl₂, 0.2 mM dNTPs, 0.5 µM of each primer, and one unit Taq DNA polymerase, using approximately 100 ng genomic DNA as template. Cycling parameters were 95°C for five minutes, followed by 35 cycles at 94°C for 30 seconds, 55°C for 30 seconds, and 72°C for 45 seconds, with a final extension step shift to the color space oligo comparison of choice. PCR products were analyzed by electrophoresis on a 1.5% agarose gel and visualized by ethidium bromide staining.

Primers were chosen from published sequences to ensure specificity, and tested in silico against GenBank sequence data to avoid cross-reaction with non-*H. pylori* *Helicobacter* species. Positive (ATCC 43504 DNA of *H. pylori*) and negative control reactions (without a template of the DNA) were included in each run.

Statistical analysis

Statistical analysis was descriptive. Exact binomial confidence intervals were used for prevalence estimates. Concordance between gastric and gallbladder positivity was recorded (expected 0). No imputation was required as there were no missing data.

Statistical analyses were conducted using IBM SPSS Statistics version 26.0 (IBM Corp., Armonk, NY) and R version 4.3 (R Foundation for Statistical Computing, Vienna, Austria). Continuous variables were assessed for normality with the Shapiro-Wilk test and summarized as mean ± standard deviation (SD) if normally distributed or median (interquartile range, IQR) otherwise; between-group comparisons used the independent samples t-test (reported with t value and degrees of freedom) or the Mann-Whitney U test (reported with U and z). Categorical variables were summarized as numbers (percentages) and compared using the χ² test (reported with χ² and df) or Fisher’s exact test when expected cell counts were <5. Prevalence estimates (e.g., gastric *H. pylori* PCR positivity) were presented with exact (Clopper-Pearson) 95% confidence intervals. Concordance between paired gastric and gallbladder results was examined with cross-tabulation; McNemar’s test (reported with χ²) was planned for paired proportions, but is not applicable when all observations fall in a single discordant cell (as occurred for gallbladder negatives). All tests were two-tailed, and statistical significance was defined a priori as p < 0.05. No imputation was required as there were no missing data. Where appropriate, effect sizes (Cohen’s d for t-tests, Cramér’s V for χ²) are reported alongside p-values to aid interpretation.

## Results

Patient demographics

The mean age of the participants was 45 ± 11.2 years. Of the cohort, 64.89% were female and 35.1% were male. Laparoscopic cholecystectomy was performed in 81.9% of patients, whereas 18.08% underwent an open procedure. Geographically, 68.08% of patients were from Karnataka, 20.21% from Andhra Pradesh, 7.44% from Tamil Nadu, and 4.25% from Kerala (Table 1).

**Table 1:** Demographic details.

Variable	Value
Total patients	49
Mean age (years)	45 ± 11.2
Female	32 (65.3%)
Male	17 (34.7%)
Laparoscopic cholecystectomy	40 (81.6%)
Open cholecystectomy	9 (18.4%)
From Karnataka	25 (51%)
From Andhra Pradesh	10 (20.4%)
From Tamil Nadu	8 (16.3%)
From Kerala	6 (12.2%)

Among the 10 patients with *H. pylori*-positive gastric biopsies (by DNA PCR), all exhibited chronic cholecystitis on histopathological examination of the gallbladder.

Gallbladder and gastric sample analysis

A total of 49 gallbladder and 49 gastric mucosal samples were analyzed. The key findings are summarized in Table 2.

**Table 2:** Summary of results. PCR: polymerase chain reaction.

Parameter tested	Result
Total gallbladder samples collected	49
Total gastric mucosal samples collected	49
Rapid urease test results (gallbladder)	All 49 negative
Rapid urease test results (gastric samples)	All 49 negative
Culture results (gallbladder)	No growth
Culture results (gastric samples)	No growth
Modified Giemsa stain (gallbladder)	Negative for *H. pylori* in all 49 samples
DNA PCR (gastric samples)	10/49 positive for *H. pylori*
DNA PCR (gallbladder samples)	All 49 were negative for *H. pylori*

Histopathology findings

Modified Giemsa staining was performed in all gallbladder tissue samples, and was negative for *H. pylori* in every case (Table 3).

**Table 3:** Histopathological examination of gallbladder specimens.

Histopathological diagnosis	Frequency (%)
Chronic cholecystitis	43 (88%)
Tubular adenoma	1 (2%)
Gangrenous cholecystitis	2 (4%)
Xanthogranulomatous cholecystitis	1 (2%)
Empyema gallbladder	1 (2%)
Eosinophilic cholecystitis	1 (2%)

Primary outcome reported 0/49 (0%) gallbladder-positive cases. Secondary outcome reported 10/49 gastric PCR-positive cases (20.4%, 95% CI: 10.2-33.7%).

## Discussion

*Helicobacter pylori*, a gram-negative, microaerophilic bacterium, is mainly linked to gastric pathologies like peptic ulcer disease and gastric malignancy. It is also associated with extragastric conditions, including autoimmune diseases and hepatobiliary disorders [8,9]. This study investigated the potential role of *H. pylori *in gallbladder disease among South Indian patients. Despite earlier studies suggesting the role of *H. pylori* in gallbladder pathology, our results showed no *H. pylori* colonization in gallbladder tissues. None of the samples were positive for *H. pylori* by RUT, culture, modified Giemsa staining, or DNA PCR. These findings align with Monstein et al. [10], who reported no *H. pylori* DNA in gallbladder specimens. While *H. pylori* was absent in gallbladder tissues, DNA PCR detected it in 20.4% of gastric biopsies. This prevalence is similar to other reports from the Indian subcontinent, with infection rates from 20% to 80% depending on the population studied [11]. The absence of *H. pylori* in gallbladder tissue, even with concurrent gastric infection, suggests gallbladder colonization by *H. pylori* is unlikely in this population.

Several mechanisms have been proposed for how *H. pylori* might contribute to gallbladder disease, including direct colonization of biliary epithelium, chronic inflammation, and altered bile composition [12,13]. However, our findings indicate these mechanisms may not be significant in South Indian patients with cholelithiasis or other gallbladder conditions. The lack of detection could be due to differences in regional bacterial strains, host immune responses, or environmental factors affecting colonization. Another factor is the technical limitations of detecting low bacterial loads in gallbladder tissue. However, the use of multiple detection methods (culture, urease test, PCR, and histology) enhances the reliability of these negative findings [14,15].

The predominance of chronic cholecystitis (88%) aligns with typical gallbladder disease pathology in symptomatic patients undergoing cholecystectomy. Notably, all patients positive for gastric *H. pylori* showed chronic cholecystitis in the gallbladder, but without detectable gallbladder colonization, suggesting an incidental rather than causative association. While our study did not detect *H. pylori* in gallbladder tissue, DNA PCR detected it in 20.4% of gastric samples. This finding contrasts with some studies from North India and Western populations, where *H. pylori* has been found in both gastric and gallbladder tissues and is associated with gallbladder cancer. For example, Apostolov et al. [16] and Nath et al. [8] reported *H. pylori* DNA in gallbladder tissue of patients with cancer and chronic cholecystitis. These discrepancies may reflect geographical, dietary, microbiological, or methodological differences and support the need for further multicentric studies with standardized testing protocols [17].

Notably, the absence of *H. pylori* in gallbladder tissue in our South Indian cohort contrasts with studies from North India, where *H. pylori* prevalence in gastric mucosa has been reported as high as 45%, and gallbladder cancer incidence is significantly higher. This regional discrepancy may be due to dietary, genetic, or environmental differences, suggesting a geographical variation in the microbiological etiology of gallbladder disease [18,19].

Strengths and limitations

This study has several key strengths. It is one of the rare studies to prospectively study the relationship between gastric and gallbladder colonization of *H. pylori* in a South Indian population. The use of several diagnostic methods (culture, RUT, histopathology, and PCR) was intended, on one hand, to improve the sensitivity of the diagnosis but also to minimize the risk of false negatives. Sterile sample retrieval during surgery kept contamination to a minimum and used specific primers with adequate controls in PCR, which made it specific. Further, the regional approach allows us to gain new insights into potential geographic variation in the *H. pylori*-gallbladder association, which is of significant clinical interest, given differences in gallbladder cancer rates between regions.

The study also has some limitations that need to be acknowledged. First, although the sample size was based on an a priori calculation, the relatively small cohort (n = 49) limits statistical power and reduces the precision of prevalence estimates, as reflected in wide confidence intervals. Rare cases of colonization may have gone undetected. Second, possible selection bias should be addressed, as we included only patients treated with cholecystectomy and a clinical indication for preoperative gastroscopy. This subgroup might not be completely representative of all patients with gallstone disease and could have an impact on the rates of gastric *H. pylori* prevalence. Third, the regional selectivity of the cohort is a strength and a limitation: its geographical setting is such that results can be interpreted only in the context of South India, but they should not be extrapolated to other inhabited regions at high risk for gallbladder cancer, such as North India.

Regarding methodological transparency, PCR protocols have been described in sufficient detail to make reproducibility possible despite the possibility that culture, RUT, and histopathology protocols could vary among laboratories. We recognize that it might not be fully reproducible by other authors following different culturing conditions, incubation settings, or diagnostic cutoffs in histology. In future multicenter studies, these methods need to be standardized for more external reproducibility. Further, targeting only *H. pylori* in our study, other *Helicobacter* species (i.e., *H. hepaticus* and *H. bilis*) may colonize the hepatobiliary tree and then play a role in disease, which were not accounted for here.

Finally, while demographic information on the study population was reported, subgroup analyses (such as male vs. female prevalence) were not thoroughly evaluated, and this is a limitation of this work. Moreover, although our study is one of the few from South India, comparison with North Indian data indicates some regional differences that need to be investigated in larger multi-regional cohorts.

## Conclusions

This study found no evidence of *H. pylori* colonization in gallbladder tissues among South Indian patients undergoing cholecystectomy, despite detecting gastric *H. pylori* DNA in 20.4% of biopsies. These findings suggest that, within this cohort, gastric *H. pylori* infection does not correlate with gallbladder colonization or pathology. Importantly, our results apply specifically to this relatively small South Indian cohort and should not be interpreted as demonstrating an absolute absence of gallbladder colonization in all populations.

When compared with studies from North India and other high-incidence regions, where both gastric *H. pylori* prevalence and gallbladder cancer rates are significantly higher, our findings highlight potential regional variation in microbial contributions to gallbladder disease. This underscores the need for multicenter and multi-regional studies across India and globally to explore how host, environmental, and microbial factors interact in gallbladder pathology. Finally, while this study focused on *H. pylori*, it is important to consider that other *Helicobacter* species (such as *H. hepaticus* or *H. bilis*) or systemic effects of gastric infection may contribute to hepatobiliary disease and were not evaluated here. Future research should expand diagnostic targets beyond *H. pylori* and employ standardized methodologies across larger cohorts to clarify the potential microbial and geographic determinants of gallbladder disease.
